# Dental Implant Materials: Current State and Future Perspectives

**DOI:** 10.3390/ma14020371

**Published:** 2021-01-14

**Authors:** Marco Cicciù, Marco Tallarico

**Affiliations:** 1 Department of Biomedical and Dental Sciences, Morphological and Functional Images, University of Messina, 98100 Messina, Italy; 2 Department of Periodontology and Implantology, University of Sassari, 07021 Sassari, Italy; me@studiomarcotallarico.it

A dental implant is a medical device used to functionally and aesthetically rehabilitate the lack of one or more teeth, allowing the support of a prosthetic substitute through direct bone support. The prerequisites for the long-term success of osseointegrated dental implants are healthy bone and soft tissues. Since both could assist with atrophy after a dental extraction procedure, it is sometimes necessary to regenerate hard and soft tissues in order to recreate ideal conditions. Before the discovery of the osseointegration principle by a Swedish school, attempts to prosthetically rehabilitate the dentition by supporting the bone bases relied on the three-dimensional shape of the endosseous section to try to obtain adequate stability against functional stress. Subperiosteal, blade, anchor, needle, disc, and numerous other types of implants were then created in the constant search for the most effective mechanical anchoring system. Nowadays, the universally adopted shape is the cylindrical or cylindrical–conical one, imitating the root of natural teeth, and resistance to functional stresses is provided by the correct osseointegration process, obtained thanks to the combination of primary stability, rigorous surgical procedures, adequate prosthetic design and realization, and correct post-operative tissue management. A traditional intervention, after carrying out the necessary preliminary assessments on the quantity and quality of the supporting bone, is performed under local anesthesia and involves the elevation of a mucoperiosteal flap to expose the bone tissue, in which deep holes are then drilled to an adequate width, where the implants or fixtures are subsequently inserted. The number and size of the implants are determined by the amount of bone available and the type of prosthetic required for the final rehabilitation. At the end of the operation, the site can be covered entirely by the sutured flap to obtain “protected” healing from bacterial and epithelial colonization. The most commonly used implant is therefore composed of an endosseous part, called a “fixture”, usually cylindrical or trunk –conical (or a combination of these shapes), threaded and equipped with other accessory retentions such as holes and grooves to increase the contact surface with the bone tissue. To provide further retention, the surfaces are treated to obtain a micrometer-scale and—more recently—nanometer-scale roughness, which has been shown to be able to actively favor the process of connection with bone tissue (osteoconduction). The material of choice used for dental implants is titanium in its commercially pure form (CP4) as a material with excellent mechanical strength and high biocompatibility characteristics [1,2]. Implants have been proposed that use zirconia also for the endosseous part as a highly biocompatible material with good mechanical characteristics, and for this reason, it is already used for the construction of orthopedic prostheses. Other favorable factors appear to be less plaque build-up and better aesthetics. A particular type of intervention developed for the rehabilitation of large areas or entire edentulous arches is that of computer-assisted implantology, which involves the preliminary simulation of a three-dimensional reconstruction of the tissues created by computer, based on Computed Tomography (CT) or cone-beam radiographs and models developed by impressions [3]. A customized surgical guide is then obtained which, fixed in the patient’s mouth, guides the implant placement surgery, greatly simplifying the procedure, which does not require exposure of the bone tissue (flapless). In many cases, a previously prepared temporary or definitive prosthetic structure is fixed at the same time as the implant insertion procedure. The long-term prognosis of dental implants can be considered reliable and predictable, now being able to rely on more than forty years of worldwide clinical experience [4,5]. The data reported in the literature show variable failure rates, depending on the surgical techniques and the types used, with poor uniformity in the selection of the parameters examined and in the duration of the observation, so it is often difficult to evaluate the different studies. Thanks to the Special Issue [6] of *Materials*, it was possible to highlight all the current conditions and the state of the art in implantology, but also all those future prospects related to new materials.

## References

[1] Makary C., Menhall A., Zammarie C., Lombardi T., Lee S.Y., Stacchi C., Park K.B. (2019). Primary Stability Optimization by Using Fixtures with Different Thread Depth According to Bone Density: A Clinical Prospective Study on Early Loaded Implants. Materials.

[2] Cicciù M. (2020). Bioengineering Methods of Analysis and Medical Devices: A Current Trends and State of the Art. Materials.

[3] Tallarico M. (2020). Computerization and Digital Workflow in Medicine: Focus on Digital Dentistry. Materials.

[4] Mastrangelo F., Quaresima R., Abundo R., Spagnuolo G., Marenzi G. (2020). Esthetic and Physical Changes of Innovative Titanium Surface Properties Obtained with Laser Technology. Materials.

[5] Beretta M., Poli P.P., Pieriboni S., Tansella S., Manfredini M., Cicciù M., Maiorana C. (2019). Peri-Implant Soft Tissue Conditioning by Means of Customized Healing Abutment: A Randomized Controlled Clinical Trial. Materials.

[6] Cicciù M., Tallarico M. Dental Implant and Materials. https://www.mdpi.com/journal/materials/special_issues/dental_implants_materials.

